# Biomonitoring of Mycotoxins: Silent Invaders and Their Subtle Assault on Human and Animal Health

**DOI:** 10.3390/toxins18080357

**Published:** 2026-08-21

**Authors:** Ana Juan-García, Cristina Juan García

**Affiliations:** Laboratory of Food Chemistry and Toxicology, Faculty of Pharmacy, University of Valencia, E-46100 Valencia, Spain

Biomonitoring is a valuable tool for assessing exposure to toxicants and understanding their potential impact on human and animal health. It involves the detection and quantification of toxicants, their metabolites, or other biomarkers in biological matrices such as tissues, secretions, excreta, or expired air [1]. This approach provides important information on internal exposure and helps to better characterize the absorption, distribution, metabolism, and elimination of toxic compounds. However, the complexity and diversity of metabolites formed during these processes require highly sensitive and selective analytical approaches capable of providing a comprehensive picture of toxicant fate within the organism.

This is particularly relevant for mycotoxins, which can be present at low concentrations in a wide variety of foods and feeds and undergo extensive biotransformation after exposure. The liver and gastrointestinal tract play key roles in their metabolism, with cytochrome P450 enzymes being among the main systems involved [1,2]. Although biotransformation generally contributes to detoxification and elimination, it may also produce metabolites with different biological properties or, in some cases, enhanced toxicity. Consequently, the characterization of mycotoxin metabolism and bioavailability is essential for accurately interpreting occurrence data and biomonitoring results and for obtaining a more realistic assessment of potential health risks.

In recent years, major advances in chromatographic and mass spectrometric technologies have considerably expanded the possibilities for biomonitoring mycotoxins and their metabolites [3]. The combination of gas or liquid chromatography with tandem and high-resolution mass spectrometry, including QTRAP-MS/MS, MS/MS, and QTOF-MS/MS platforms, provides the sensitivity, selectivity, and structural information required for their detection at trace levels. These developments have strengthened our ability to investigate mycotoxin biotransformation and bioavailability, contributing to a deeper understanding of exposure and toxicity and supporting more accurate risk assessment.

This book explores the complex and multifaceted landscape of mycotoxin contamination, moving from advanced detection methods in medicinal plants to the molecular mechanisms of neurotoxicity, prenatal vulnerabilities, and large-scale public health interventions. In it, five chapters synthesize current research to provide a comprehensive understanding of these fungal metabolites and their impact on human health.

In Chapter 1, Huang et al. (Contribution 1) deal with high-throughput detection and risk in dual-purpose plants. They address the urgent need for sophisticated analytical tools to monitor multi-mycotoxin contamination in materials that serve both as food and medicine. This chapter details the development of an optimized QuEChERS pretreatment method coupled with LC-MS/MS to simultaneously detect 73 different mycotoxins. Focusing on lotus seed, coix seed, licorice root, and dried tangerine peel, the research reveals that multi-mycotoxin co-occurrence is widespread, with seed-type plants showing particular susceptibility to *Aspergillus* and *Fusarium* species. By applying Margin of Exposure (MOE) and Hazard Index (HI) models, this chapter establishes a reference for monitoring plant-based matrices and emphasizes the critical role of moisture control and intact storage in reducing health risks.

Moving from occurrence to cellular impact, Chapter 2, by Moyano-Lopez et al. (Contribution 2), deals with neurotoxicity and the complexity of binary mixtures of mycotoxins. It investigates the cytotoxic profiles of Beauvericin (BEA), Citrinin (CTN), Moniliformin (MON), and Patulin (PAT). While many studies focus on single toxins, this work highlights the realistic scenario of co-exposure. Using the SH-SY5Y human neuroblastoma cell line, the chapter demonstrates that toxicity is not always additive; for instance, while PAT shows high individual toxicity, certain binary combinations like BEA + MON and CTN + PAT reveal enhanced cytotoxic effects that could endanger neuronal health. These findings underscore the necessity of expanding regulatory frameworks to account for the synergistic interactions of mycotoxin mixtures.

Chapter 3, from García-Esparza et al. (Contribution 3), addresses ochratoxin A and the breach of biological barriers. A specialized focus is placed on Ochratoxin A (OTA), a potent toxin that transcends its well-known role in kidney damage to target the central nervous system (CNS). This systematic review explores the mechanisms by which OTA “loosens” tight junctions to cross the intestinal and blood–brain barriers (BBB). Once within neural structures, OTA triggers neuroinflammation, apoptosis, and neurogenesis defects by activating microglial cells and astrocytes. The chapter also evaluates the protective role of antioxidant systems, such as glutathione and polyphenols, in neutralizing OTA-induced oxidative stress, offering insights into potential neuroprotective strategies against neurodegenerative diseases.

In Chapter 4, from Aragao et al. (Contribution 4), the focus shifts to the most vulnerable populations, based on prenatal exposure and developmental vulnerability. It presents the first Brazilian study quantifying mycotoxin biomarkers directly in fetal and neonatal tissues. By analyzing liver and serum samples from stillborns and neonates, researchers confirmed the effective transplacental transfer of major toxins, including AFM1, OTA, and ZEN. The frequent detection of mycotoxin co-occurrences in these samples suggests a troubling link between maternal dietary exposure and congenital anomalies, prematurity, and intrauterine growth restriction (IUGR). This chapter advocates for proactive risk assessment and dietary guidance during pregnancy to safeguard early human development.

Finally, Chapter 5, from Chen et al., synthesizes five decades of research in China regarding the link between Aflatoxin B1 (AFB1) and hepatocellular carcinoma (HCC) (Contribution 5). This chapter details the synergistic interaction between AFB1 and chronic Hepatitis B (HBV), anchored by the detection of the TP53 R249S molecular fingerprint. It reviews the success of “natural experiments,” such as the dietary shift from maize to rice, which led to a sustained decline in HCC mortality. To conclude, the chapter proposes an integrated “5 + 1” prevention framework—spanning source control, tiered governance, and climate-sensitive early warning systems—as a global template for managing mycotoxin risks in a changing environment.
